# Epigenetics in diabetic cardiomyopathy

**DOI:** 10.1186/s13148-024-01667-1

**Published:** 2024-04-05

**Authors:** Xiaozhu Ma, Shuai Mei, Qidamugai Wuyun, Li Zhou, Dating Sun, Jiangtao Yan

**Affiliations:** 1 Division of Cardiology, Department of Internal Medicine, Tongji Hospital, Tongji Medical College, Huazhong University of Science & Technology, Wuhan, China; 2 Hubei Key Laboratory of Genetics and Molecular Mechanisms of Cardiological Disorders, Wuhan, China; 3 Department of Cardiology, Wuhan No. 1 Hospital, Wuhan Hospital of Traditional Chinese and Western Medicine, Wuhan, China; 4 Genetic Diagnosis Center, Tongji Hospital, Tongji Medical College, Huazhong University of Science and Technology, Wuhan, China

**Keywords:** Diabetic cardiomyopathy, Epigenetic regulation, DNA methylation, Histone modification, Non-coding RNA

## Abstract

Diabetic cardiomyopathy (DCM) is a critical complication that poses a significant threat to the health of patients with diabetes. The intricate pathological mechanisms of DCM cause diastolic dysfunction, followed by impaired systolic function in the late stages. Accumulating researches have revealed the association between DCM and various epigenetic regulatory mechanisms, including DNA methylation, histone modifications, non-coding RNAs, and other epigenetic molecules. Recently, a profound understanding of epigenetics in the pathophysiology of DCM has been broadened owing to advanced high-throughput technologies, which assist in developing potential therapeutic strategies. In this review, we briefly introduce the epigenetics regulation and update the relevant progress in DCM. We propose the role of epigenetic factors and non-coding RNAs (ncRNAs) as potential biomarkers and drugs in DCM diagnosis and treatment, providing a new perspective and understanding of epigenomics in DCM.

## Introduction

According to the International Diabetes Federation, the population with diabetes is estimated to reach 600 million by 2045 [1]**,** posing a critical threat to the health and safety of individuals and causing a heavy burden on medical care worldwide. Patients with diabetes primarily develop cardiovascular complications, which are the primary contributors to mortality. Diabetic cardiomyopathy (DCM), defined by Rubler et al. in 1972 [2], is a clinical condition caused by abnormal glycolipid metabolism that develops into heart failure without coronary heart disease, hypertension, or valvular disease [3]. As a typical metabolic cardiomyopathy, it includes the early subclinical period, which manifests as diastolic dysfunction, characterized by cardiac hypertrophy and myocardial fibrosis, and evolves to systolic dysfunction accompanied by heart failure with reduced ejection fraction [4]. Emerging studies have shown that patients with diabetes have a three to five times greater risk of adverse cardiovascular events than those without the disease [5]. Unfortunately, there are no specific drugs targeting the pathological mechanism of DCM.

Continuous impairment and cascade reactions induced by hyperglycemia and insulin resistance cause irreversible cardiac damage owing to a combination of genetic and environmental factors [6]. Researchers have attempted to elucidate the underlying mechanisms of DCM, which are usually treated as determinants of uncontrollable persistent pathological changes, such as aberrant hyperglycemia, insulin resistance, excessive oxidative stress, inflammatory response, and mitochondrial dysfunction [7–9]. However, the pathological mechanisms involved in the pathophysiology of DCM have not yet been fully elucidated.

Epigenetics is a heritable and invertible pattern without alterations in the DNA sequence and is closely related to environmental stimulations [10]. When first proposed by Waddington in 1942 [11], it had attracted researchers and has been applied to elucidate the underlying pathophysiological processes. Epigenetics has expanded the understanding of the fundamental pathological changes in biological development. Several studies have confirmed that epigenetic regulation is involved in the development of various diseases, particularly cardiovascular diseases. Given the genetic and environmental factors involved in the progression of DCM, we believe that fully elucidating the mechanisms underlying the pathogenesis of DCM based on epigenetic regulation will provide strong support for exploring effective therapeutic drugs. Recently, an increasing number of epigenetic regulatory mechanisms have been investigated with the development of sequencing technology. In this review, we focus on the advanced epigenetic progress in DCM to provide scientific and theoretical support for identifying novel potential intervention targets for clinical translation.

## Overview of epigenetics

**Fig. 1:**
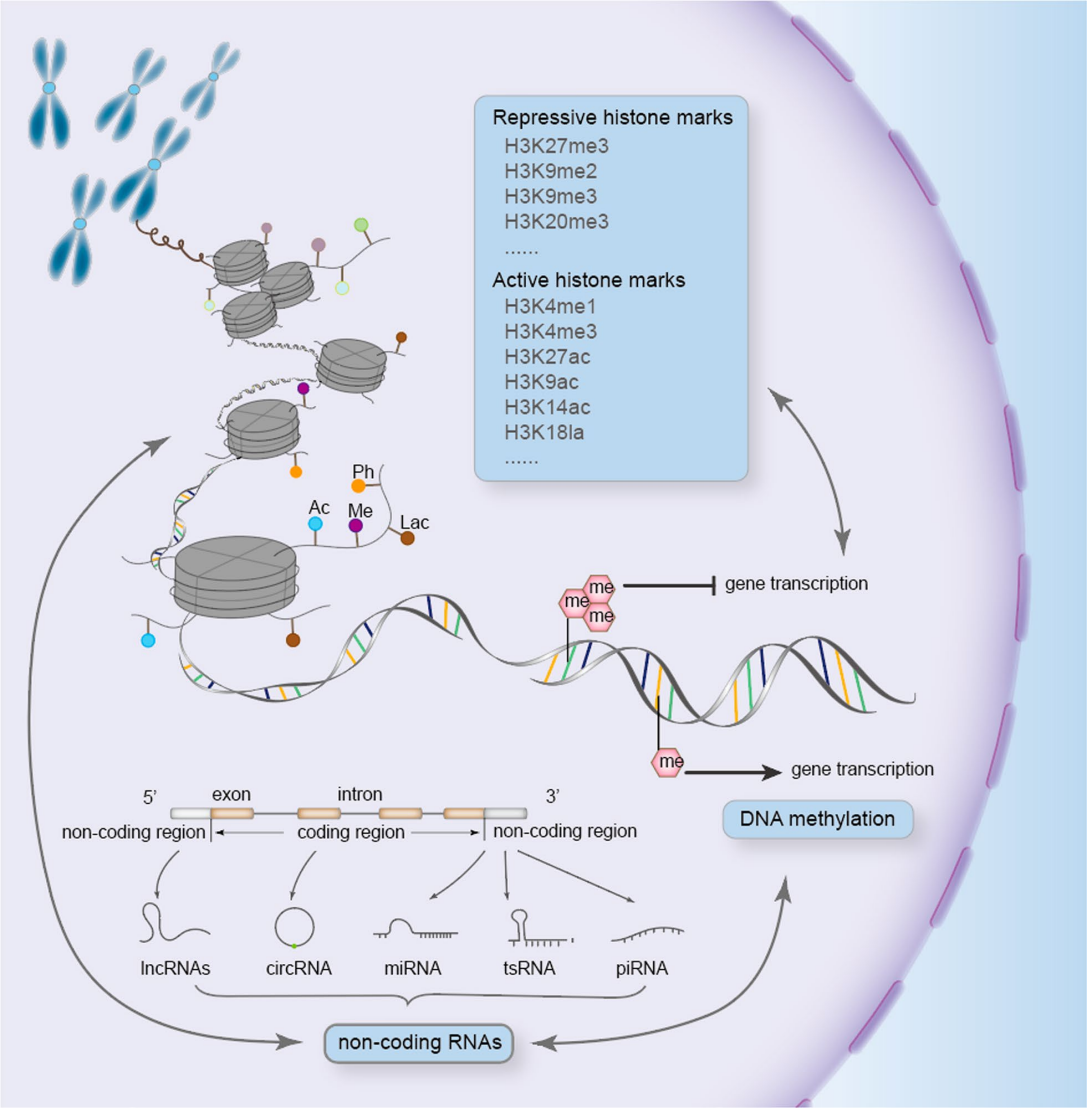
Diagrammatic representation of three main epigenetic models. The gene expression could be modulated at multiple levels, including histone modifications, DNA methylation, and non-coding RNAs. Briefly, the histone post-translational modifications are categorized as two types. Repressive histone modifications including H3K27me3 and H3K9me3 mainly distributed in the heterochromatin region, where the chromatin structure is tight. Active histone modifications are divided into H3K4me1, H3K4me3, and H3K27ac. They are mainly distributed in the autochromatin region, which is more conducive to the gene transcription. DNA methylation on CpG islands plays different roles in gene expression depending on the number of methyl as well as the modification sites. Various non-coding RNAs generated by transcription of non-coding regions also regulate gene expression in the nucleus or cytoplasm at transcriptional or post-transcriptional levels

Epigenetic regulation serves as a bridge between the environment and heritable disease phenotypes. The fundamental modes of epigenetic regulation can be classified into three types: DNA methylation, histone modification, and non-coding RNA (Fig. 1).

### DNA methylation

DNA methylation was the earliest well-studied pattern of epigenetic modification in the 1960s [12], typically occurring on the fifth carbon atom of cytosine (5mC). DNA modification is associated with many cellular biological processes, such as transcriptional regulation, genomic imprinting, and X-chromosome inactivation.

The effects of DNA methylation on gene activity primarily depend on different genomic regions, including CpG islands, intergenic regions, and genomic regions. Genome-wide analysis has shown that CpG islands are present in 60% of the promoter regions of the human genome [13], suggesting that dynamic changes in DNA methylation influence gene transcription and may play a role in growth and development. Hypermethylation at the CpG island recruits repressive methyl-modulating factors and contributes to maintaining heterochromatin status. Therefore, DNA methylation inhibits gene expression. Similarly, in the intergenic regions, the expression of non-coding gene elements is negatively correlated with DNA methylation [14]. However, a few studies have shown that a high level of DNA methylation in the gene is associated with increased gene expression [15].

The status of total DNA methylation is regulated by three regulators: reader, writer, and eraser, which identifies, catalyzes, and removes, respectively. Transcription factors with sequence-dependent mCpG-binding activity bind to specific sequences, initiating the methylation process [16]. The production and maintenance of DNA methylation highly depend on three DNA methyltransferases (DNMTs) with different functions: Dnmt1, Dnmt3a, and Dnmt3b. Dnmt3a and Dnmt3b catalyze the unmodified DNA chain and mediate de novo methylation, whereas Dnmt1 participates in DNA replication and repair by methylating hemimethylated DNA [17, 18]. Removal of DNA methylation is mediated by the ten-eleven translocation (TET) enzyme families, including tet1, tet2, and tet3. The Dnmt and Tet families are closely associated with multiple cardiovascular diseases under various pathological conditions and environmental stress [19]. Dnmt3a/3b protein levels in the myocardium are reduced during the development from fetal to adult stages but are reactivated in transverse aortic constriction-induced cardiac hypertrophy due to increased CpG methylation in the myh6 promoter region [20]. CRISPR-Cas9-mediated Dnmt3a knockout in mice was found to aggravate severe cardiac dysfunction and fibrosis, and Dnmt1 participated in anti-apoptotic signaling pathways by regulating cardiac-specific gene methylation in the promoter [21]. Erasers such as tets promote cardiomyocyte differentiation at the cardiac progenitor stage during mouse and human cardiac development by deactivating the Wnt signaling pathway [22, 23].

### Histone modifications

A vast amount of genetic information can be preserved and precisely regulated by the folded and supercoiled chromatin structures in cells. Nucleosomes contain five types of conserved histones (H1, H2A, H2B, H3, and H4) and spiral DNA of approximately 146 bp, which is the basic structure of eukaryotic chromatin. Various post-translational modifications, such as acetylation, methylation, phosphorylation, ubiquitination, phase polymerization, and ADP ribosylation, occur at the tail or acid pocket of histones, particularly of H3 and H4, which regulate gene expression by altering chromatin accessibility.

The modulating patterns of histone modifications are classified as activating and inhibitory histone modifications according to the regulatory effects of the process on gene expression. Lysine acetylation is usually associated with gene activation, particularly at histone H3 lysine 27. Histone H3 lysine 27 acetylation (H3K27ac) significantly loosens the folded and supercoiled structure of chromatin, which is beneficial for recruiting various transcription factors and coactivators to gene promoters, enhancing gene transcription [24, 25]. Owing to its significant effect on enhancing gene transcription, H3K27ac is considered a molecular marker of super-enhancers. Trimethylated histone H3 at lysine 27 (H3K27me3) is a typical repressive histone modification that compresses the chromatin to suppress gene transcription. The dynamic balance between the two types of histone modifications in chromatin determines disease progression. Professor C. David Allis proposed the histone code hypothesis, which states that the crosstalk between different histone modifications amplifies gene-modulating signals, leading to a greater effect on the chromatin structure of target genes. This has gained increasing attention from researchers.

Notably, multiple crucial molecules combine to maintain a balance in the regulatory network of histone modifications. For lysine acetylation, histone acetyltransferases (HATs) and histone deacetylases (HDACs) catalyze the acetylation and deacetylation of phosphorylated RNA Pol II, respectively [26]. Various studies have indicated those both are critical in cardiac pathological processes, such as myocardial hypertrophy, cardiac fibrosis, endothelial hyperplasia, and smooth muscle cell migration [27–32]. Bromodomain protein 4 (BRD4) is a well-known member of the bromodomain and extra-terminal domain (BET) family. As a vital transcriptional activator and regulator, BRD4 specifically recognizes histone acetylation sites via its bromine domain, recruits many transcription complexes, and promotes acetylation [32].

Given the central role of BRD4 in gene activation in tumors and heart failure, researchers have attempted to develop its inhibitors and targets, such as JQ1, which has been widely used in basic research and clinical trials. Similarly, for lysine methylation, there are numerous types of histone methyltransferases and demethylases (KDM family, comprising the LSD family with flavin adenine dinucleotide-dependent monoamine oxidases (MAO) and another family with Fe- (II) and α-ketoglutarate-dependent dioxygenases), to maintain chromatin homeostasis. Disruptor of telomeric silencing 1-like has been reported to regulate H3K79me2 of core transcription factors-nuclear factor kappa-B (Nf-κB), mediating inflammation in atherosclerosis development [33]. The widely studied methyltransferase, enhancer of zeste homolog 2 (EZH2, the subunit of multi-comb inhibitory complex 2 [PRC2]), whose inhibitors have been used in tumor therapy, participates in gene expression silencing by catalyzing H3K27me3 [34]. In addition, the switch from EZH2 to EZH1 reportedly mediates cardiac regeneration [35].

### Non-coding RNAs

Even with the rapid development of high-throughput technology, scientists were surprised to discover that less than 2% of transcripts have protein-encoding potential in the human genome [36]. Several non-coding RNAs (ncRNAs) are considered to be gene-expressive noise and participate in regulating gene expression.

ncRNAs are usually classified into long non-coding RNA (lncRNAs) and small non-coding RNAs according to their sequence length. lncRNAs are long ncRNAs with lengths of > 200 nt, some of which can encode short peptides. They are less conserved across species and are highly cell-type-specific. Their functions are complex and are associated with their location. Diverse modulation models have been reviewed by Mendell et al. [37]. Nuclear lncRNAs are involved in many processes, such as chromatin dynamics and RNA splicing, by recruiting transcription factors or binding to transcriptional regulatory complexes, whereas cytoplasmic lncRNAs act as scaffolds for chromatin remodeling complex combinations or microRNA (miRNA) sponges to participate in mRNA transport and protein stability. Small non-coding RNAs can be further classified as miRNAs, circularRNAs, tRNA-derived small RNAs, and PIWI-interacting RNAs. Unlike lncRNAs, small non-coding RNAs are relatively conserved. miRNAs recruit miRNA-induced silencing complexes (MiRISCs) and bind to the 3' untranslated region (UTR) of their target genes, inhibiting gene translation. Similarly, nuclear miRNAs modulate target genes at the transcriptional level by binding to the promoters of the target gene.

Epigenetic regulation modes do not exist independently; they rather have interrelated influences, forming a complex regulatory network that collectively maintains the epigenetic regulatory homeostasis of genes. For example, a typical lncRNA-Hotair has been investigated to be related to multiple cardiovascular diseases. A previous study showed that Hotair could recruit the histone modification writer PRC2, affecting cell proliferation, differentiation, and metabolism by changing chromatin structure [38]. In addition, Hotair could bind to miRNA 331-3p as a competitive endogenous RNA and participate in tumor metastasis [39]. The scope of miRNA regulation is broad, given that small molecules and more than 30% of genes in the human genome are targeted and regulated by miRNAs. Multiple studies have shown that miRNAs target genes that encode histone-modifying enzymes, such as HDAC, DNMT, and EZH, thus highlighting the relationship among ncRNAs, DNA methylation, and histone modification [40, 41].

## Epigenetic regulation in DCM

As a metabolic disease, DCM is susceptible to environmental factors, such as glycolipid homeostasis which vastly influences epigenetic states. Therefore, it is likely that epigenetic regulation plays a critical role in DCM. Based on the current researches, we describe the role of epigenetic regulations in DCM and provide new insights into the pathogenesis and treatment of DCM.

### DNA methylation in DCM

DNA methylation is highly related to diabetic status and is crucial in vital pathological processes in DCM [42]. Various signaling pathways are activated under different levels of methylation, which occurs in the promoter regions of multiple metabolic genes.

JunD is a member of the AP-1 transcription factor family that is involved in cardiac aging, angiogenesis, and metabolic processes [43, 44]. Hussain et al. found that JunD expression was reduced in the heart tissues of patients with diabetes and DCM mice [45]. Cardiac-specific JunD overexpression ameliorated cardiac dysfunction by mitigating oxidative stress, inflammatory responses, and cardiac impairment in DCM. Various epigenetic modifications regulate JunD expression. Quantitative polymerase chain reaction was used to confirm that methylation of the JunD promoter region was up-regulated in diabetic hearts. Furthermore, DNA methylation-induced repressive epigenetic modifications, such as H3K9me3 and multiple endocrine neoplasia 1, were up-regulated. These results indicated that hyperglycemia-induced hypermethylation of the JunD promoter compressed the chromatin structure and inhibited JunD transcription.

Calcium imbalance is a pathological mechanism of DCM. As a transmembrane transporter, sarcoplasmic/endoplasmic reticulum Ca^2+^ ATPase 2a (SERCA2a) is primarily distributed in cardiomyocytes and assists in transferring Ca^2+^ ions from the cytoplasm to sarcoplasmic reticulum, thereby maintaining calcium homeostasis in cardiomyocytes. Studies have shown that it significantly affects the development of various diabetic complications, particularly DCM [46, 47]. An early study showed that the methylation level of the SERCA2a promoter region was enhanced under tumor necrosis factor-alpha stimulation, and reduction of SERCA2a exacerbated calcium imbalance and oxidative stress in cardiomyocytes.

Glutathione peroxidase 1 (GPX1) is an antioxidant enzyme involved in DCM development; it reduces the production of reactive oxygen species (ROS) in cardiomyocytes and improves insulin resistance [48]. Given that DNMTs are associated with glycolipid and energy metabolism, several researchers have investigated their roles in DCM [49–51]. Many advanced glycation end products are produced in diabetic environments, which promote the methylation of the GPX1 promoter region, thereby aggravating oxidative stress and apoptosis in cardiomyocytes. Zhu et al. identified the exact enzyme that mediates the methylation process and found that DNMT2 is crucial in GPX1 expression [50]. Dnmt1, Dnmt3a, and Dnmt3b expression levels are downregulated in Akita diabetes. The suppressor of cytokine signaling (SOCS)1/3 promoter methylation is increased, and SOCS1/3 activates the JAK-STAT signaling pathway in hepatocytes and stimulates the transcription of insulin-like growth factor 1, which mediates oxidative stress in diabetic cardiomyocytes [52]. In addition, the renin–angiotensin–aldosterone system is overactivated during the development of DCM, inducing ventricular hypertrophy and remodeling. Studies have shown that the gene expression of angiotensin receptor 1b highly depends on the methylation level of its promoter [53]. Hypoxia-inducible factor methylation is associated with glucose metabolism and insulin sensitivity, which are the primary factors involved in DCM development. In a recent case–control study, HIF-3A levels decreased in the peripheral blood of patients with DCM [54]. Moreover, HIF3a mRNA expression and the intron 1 methylation rate were negatively correlated.

### Histone modifications in DCM

Histone acetylation is involved in the pathogenesis of coronary artery disease, hypertension, arrhythmia, and heart failure and is the most studied form of histone modification in DCM [55].

Nicotinamide adenine dinucleotide (NAD +) -dependent sirtuin is a highly conserved class III deacetylase that targets the covalent modification of lysine at specific histone sites, exerts its epigenetic regulatory role, and acts as a transcription factor to modulate gene expression, thereby participating in various pathological processes of DCM, such as oxidative stress, inflammation, cell differentiation, mitochondrial metabolism [56–58]. Palomer et al. [59] reviewed the multiple functions of SIRT in DCM pathophysiology. A recent study indicated SIRT3 could mediate mitochondrial translation and protest against diabetes-induced cardiac dysfunction by reducing Ago2 malonylation from a new perspective [60]. Chen et al. found that HDAC inhibition attenuated cardiac hypertrophy and interstitial fibrosis in a streptozotocin (STZ)-induced diabetic model by increasing acetylated GLUT1 and phosphorylated p38 expression [61]. Additionally, HDAC inhibition reportedly had a cardioprotective effect within a short period of hyperglycemia treatment. Furthermore, Xu et al. found that HDAC3 appeared to be the most effective subtype [62]. In this study, the cardiac function of DCM mice treated with the specific HDAC3 inhibitor, RGFP966, was better than that of those treated with the pan-HDAC inhibitor, valproic acid. Previous studies indicated that diabetes results in impaired proliferation and reprogramming of cardiac-specific mesenchymal cells. Global histone code profiling of cardiac mesenchymal stem cells from patients with diabetes was performed to analyze epigenetic alterations, and the results indicated that H3K9Ac and H3K14Ac levels were decreased, while H3K9me3 and H3K27me3 levels were increased. Similarly, levels of some cardiac epigenetic enzymes, such as histone demethylase jmjd3, acetylase GCN5, and HAT activator SPV106, were significantly altered under diabetic conditions. These results indicated that epigenetic modifications of histones and chromatin remodeling are involved in diabetes-associated cardiac mesenchymal cell reprogramming [63, 64]. Endothelial progenitor cell-derived extracellular vesicles initiate cardiac repair mechanisms after myocardial infarction. However, Huang et al. found that reparative function was impaired in patients with diabetes and mice, suggesting that hyperglycemia aggravates cardiac dysfunction induced by ischemia–reperfusion injury [65]. Further exploration showed that endothelial gene transcription was inhibited by HDAC under diabetic conditions.

**Fig. 2:**
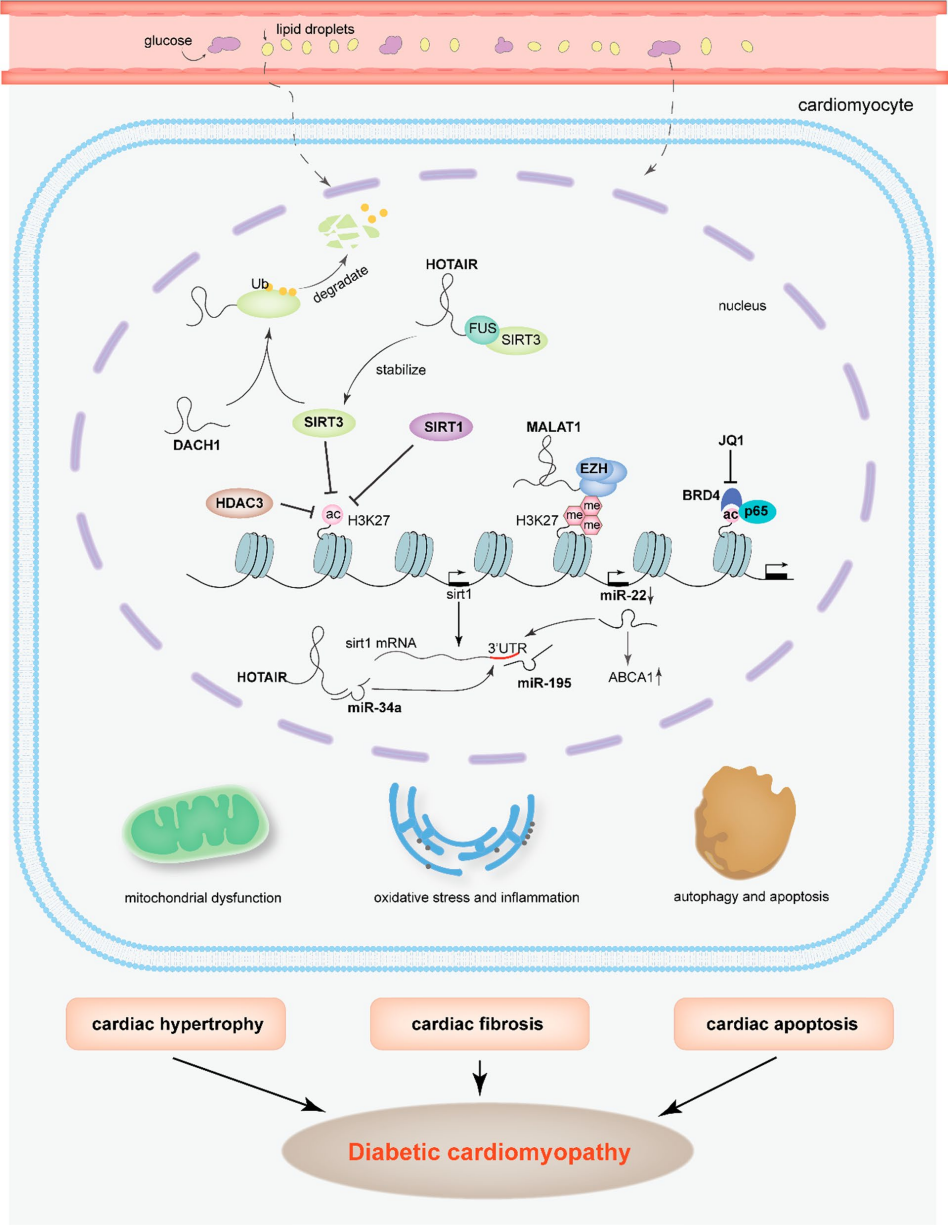
Dynamics of non-coding RNAs interacting with histone modification in diabetic cardiomyopathy. Complex epigenetic crosstalk contributes to the progress of diabetic cardiomyopathy under the condition of hyperglycemia and hyperlipidemia. lncRNA DACH1 binds to deacetylase SIRT3, accelerating its ubiquitination-dependent degradation. However, lncRNA Hotair interacts with FUS and stabilize SIRT3 indirectly. The transcription of another deacetylase, SIRT1 is regulated by various miRNAs. miR-22, miR34a, and miR195 could bind to the 3’UTR region of sirt1 mRNA. In addition, MALAT recruits EZH which hold the methyltransferase activity, inhibiting miR-22 transcription by reducing H3K27me3. BRD4 as a coactivator of p65 identifies and activates inflammatory genes, playing a crucial role in inflammation and oxidative stress. With the combination of genetic and epigenetic factors, characteristic pathological changes such as hypertrophy, fibrosis, and apoptosis occur in the heart, resulting in diabetic cardiomyopathy ultimately

In addition to epigenetic modifier enzymes, some transcriptional regulators are reportedly involved in the progress of DCM (Fig. 2). Studies have shown that BRD4 is closely associated with metabolic diseases [66]. BRD4 binds to the promoters of multiple metabolic genes and regulates cardiac fibrosis and oxidative phosphorylation [67]. The activated NF-κB signaling pathway is an essential inflammation reflection pathway in DCM. BRD4 acts as a transcriptional coactivator of p65 to mediate NF-κB-induced gene transcription in β-cells [68].

Histone lactation is a novel epigenetic reprogramming pattern discovered and proposed by Zhao et al. in 2019 [69]. Lactic acid was found to act as a precursor to lactylate histones and not just as an energy substrate. Accumulating evidence indicates that histone lysine lactylation mediates various pathological progressions in cardiac disease, such as early repair of post-myocardial infarction and mitochondrial pyruvate carrier [70–72]. A recent study showed that α-myosin heavy chain (α-MHC) K1897 lactylation was significantly reduced in AngII-induced heart failure mice owing to decreased lactation [73]. The mutation on specific lactylate sites led to a weaker α-MHC–titin interaction and induced cardiac dysfunction. Furthermore, the formation and decomposition of histone lactylation are mediated by acyltransferase p300 and delactylase SIRT1, respectively. A recent study also demonstrated that deacetylase HDAC1-3 is involved in eliminating histone lactylation [74]. These results suggest a potential association between lactylation and cardiac metabolism and a crosstalk between different histone modifications. Studies on the role of histone lactation in the progression of DCM remain limited; however, it is worth anticipating that a potential epimetabolic code based on glycolytic products is a promising prospect.

### ncRNAs in DCM

#### lncRNAs in DCM

The inflammatory response is considered an exacerbating factor in DCM development, which contributes to oxidative stress and apoptosis (Table 1) [75]. Nucleotide-binding and oligomerization domain (NOD)-like receptor thermal protein domain-associated protein 3 (NLRP3) is a cytosolic immune factor that assembles signaling complexes under various pathological conditions, such as metabolic abnormalities, mitochondrial dysfunction, aging, and environmental factors, mediating the activation of inflammatory reactions and cell death [76, 77]. NLRP3 can be activated upon hyperglycemia and hyperlipidemia stimulation and promotes the generation of pro-inflammatory factors, such as interleukin (IL)-1β, IL-6, and IL-18. Activated inflammatory factors induce apoptosis and pyroptosis, which aggravate the progression of DCM. Meng et al. found that lncRNA TINCR was significantly up-regulated in an STZ-induced DCM rat model, promoted cardiomyocyte pyroptosis, and aggravated cardiac dysfunction [78]. They further found that TINCR interacted with NLRP3 and stabilized NLRP3 mRNA, thereby accelerating the initiation and progression of DCM. lncRNA MALAT1-NLRP3 axis reportedly participated in various diabetic complications. In diabetic hearts, increased MALAT1 expression was found to aggravate cardiac pyroptosis and fibrosis. A previous study indicated that the protective effect of pomegranate peel extract on DCM relied on NLRP3/caspase-1/IL1β signaling pathway inhibition via the repression of MALAT1 expression. This suggested that MALAT1 could be a novel therapeutic target for DCM [79]. Another important lncRNA, GAS5, has been identified to be associated with metabolic disease by regulating NLRP3 [80]. The expression of GAS5 was reduced in high-fat diet-fed mice and involved in nonalcoholic fatty liver disease via NLRP3-mediated pyroptosis. In a study by Xu et al., lncRNA GAS5 expression decreased in STZ-induced DCM mouse hearts and high glucose-treated HL-1 cells. Moreover, downregulation of GAS5 promoted NLRP3 inflammasome activation and cardiac pyroptosis and exacerbated the development of DCM [81]. GAS5 acts as an miRNA-34b-3p sponge to enhance the expression of the aryl hydrocarbon receptor, negatively regulating the NLRP3 inflammasome.

**Table 1:** lncRNAs associated with diabetic cardiomyopathy

Name	Experimental animals	Expression	Pathological process	Mechanism	References
DCRF	Rat	Up	Autophagy and myocardial fibrosis	Sponging mir-551b-5p to increase pcdh17 expression	[110]
DACH1	Mouse	Up	Mitochondria, oxidative stress, cell apoptosis, fibrosis and hypertrophy	Binding to sirtuin3 and promoting sirtuin3 degradation	[111]
TINCR	Rat	Up	Pyroptosis	Promoting the expression of nlrp3 by sustaining mrna stability	[78]
Kcnq1ot1	Mouse	Up	Fibrosis and pyroptosis	Regulate the expression of caspase-1 by sponging mir-214-3p	[82]
	Mouse	Up	Apoptosis and inflammation	Sponging mir-181a-5p and increasing pdcd4 expression	[112]
ZNF593-AS	Mouse	Down	Apoptosis and inflammation	Interacted with irf3 and suppressing fatty acid-induced phosphorylation	[113]
MALAT1	Rats	Up	pyroptosis and fibrosis	–	[79]
	Rats	Up	inflammation	–	[114]
	Rat	Up	Cell scorch death	Inhibiting nlrp3 expression	[115]
	Mouse	Up	Apoptosis	Recruiting ezh2 to the mir-22 promoter region	[116]
	Rat	Up	apoptosis	/	[117]
Airn	Mouse	Down	Cardiac fibrosis	Binding to imp2 and preventing its degradation	[118]
ZFAS1	Mouse	Up	Ferroptosis and apoptosis	Sponges miR-150-5p to inhibit CCND2 expression	[119]
H19	Rat	Up	Oxidative stress, inflammation and apoptosis	Increasing miR-675 expression and further reduce VDAC1	[87]
	Rat	Up	Apoptosis	Inducing miR-29c expression and promoting MAPK13	[88]
GAS5	Mouse	Down	Inflammation and pyroptosis	Sponging miR-34b-3p and suppressing NLRP3 inflammasome activation-mediated pyroptosis	[81]
	–	Down	Apoptosis	Sponging miR-320-3p to modulate the apoptosis of NMC	[120]
	Rat	Down	Autophagy	Sponging miR-221-3p to upregulate p27	[121]
	Rat	Down	Proliferation and apoptosis	Targeting miR-138 to down-regulate CYP11B2 and attenuating cardiomyocyte injury	[122]
HOTAIR	–	Down	Pyroptosis and inflammation	Recruiting FUS to regulate SIRT3 expression	[85]
	Mouse	Down	Oxidative stress and inflammation	Sponging miR-34a to regulate SIRT1	[86]
TINCR	–	Down	Apoptosis	–	[123]
Crnde	Mouse	Up	Fibrosis	Binding to Smad3 to attenuate cardiac fibrosis	[84]
NEAT1	Rat	Down	Apoptosis	Regulating Nrf2 expression by sponging miR-23a-3p	[124]
MIAT	Rat	Up	Apoptosis	Sponging miR-22-3p to upregulate DAPK2	[125]
NORAD	Mouse	Up	Fibrosis and inflammation	Sponging to adsorb miR-125a-3p, and regulating Fyn	[126]
AK081284	Mouse	Up	Fibrosis	Promoted the production of collagen and TGFβ1	[83]
ANRIL	Rat	Up	Oxidative stress, apoptosis, inflammation, fibrosis	–	[127]
NONRATT007560.2	–	Up	Oxidative stress and apoptosis	–	[128]
MEG3	–	Up	Apoptosis	Sponging microRNA-145 to up-regulating the expression of PDCD4	[129]
PVT1	–	Up	Apoptosis	Sponge miR-23a-3p to increase CASP10 expression	[130]

Cardiac fibrosis is the characteristic feature in the advanced and late stages of DCM progression, in which the transforming growth factor beta (TGF-β)-SMAD signaling pathway is vital. Activated TGF-β triggers cellular profibrotic responses and further causes collagen deposition, cardiac remodeling, and stiffing. The lncRNA Kcnq1ot1 was activated in the cardiac tissue of an STZ-induced diabetic model compared with that in control C57BL/6 mice, suggesting that Kcnq1ot may be involved in the progression of DCM [82]. Functional in vivo and in vitro experiments using short hairpin RNA or small interfering RNA indicated that the systolic and diastolic functions of the diabetic heart were improved after silencing Kcnq1ot1, which manifested as reduced myocardial mass, inflammation, and fibrosis-related gene expression. Further, the TGF-β1/SMAD signaling pathway was markedly inhibited with Kcnq1ot1 knockdown and was rescued with repressed miR-214-3p, suggesting that lncRNA Kcnq1ot1 functioned as a miRNA sponge to regulate TGF-β1/SMAD pathway. Another lncRNA, AK081284, was proven to be associated with cardiac interstitial fibrosis via TGF-β1. Zhang et al. discovered that AK081284 mediated the effect of IL-17 on interstitial fibrosis in the diabetic heart [83]. Following AK081284 knockdown in cardiac fibroblasts, there was a reduction in mRNA expression of TGF-β and collagen synthesis genes. In addition, the lncRNA Crnde, which was primarily increased in CFs with TGF-β stimulation, was found to exert a protective effect against cardiac fibrosis. In addition, Crnde inhibited the transcriptional regulation of SMAD3 by binding to SMAD3 directly, forming a negative feedback loop between Crnde and SMAD3 [84].

Furthermore, some other lncRNAs are found to be associated with inflammation, apoptosis, and autophagy during the progression of DCM. The lncRNA HOTAIR ameliorates high glucose (HG)-induced pyroptosis and inflammation by recruiting the fused in sarcoma (FUS) protein and promoting SIRT3 expression [85]. In contrast, HOTAIR functions as a miRNA-34a sponge involved in oxidative stress [86]. Increased H19 expression under HG conditions inhibits apoptosis and inflammation by binding to different miRNAs [87, 88].

#### miRNAs in DCM

The widespread regulation of miRNAs during the development of DCM has gradually been revealed with advances in sequencing technology (Table 2). Different miRNA expression profiles have been reported at different stages of DCM [89].

**Table 2:** miRNAs related to diabetic cardiomyopathy

Name	Experimental animals	Expression	Pathological process	Target genes	References
miR-320	Mouse	Up	Hyperlipidemia and hyperglycemia	cd36	[95]
miR-207	Mouse	Up	Autophagy	lamp2	[131]
miR-30d	Rat	Up	Pyroptosis, inflammation and apoptosis	foxo3a	[132]
			Autophagy	klf9/vegfa	[133]
miR-223	Rat	Up	Inflammasome activation, fibrosis, and apoptosis	–	[134]
	Mouse	Up	Glucose metabolism	glut4	[135]
miR-29	Mouse	Up	Fibrosis	–	[136]
miR-150	–	Up	Inflammation and fibrosis	smad7	[137]
miR-451	Mouse	Up	Lipid accumulation	cab39	[138]
miR-195	Mouse	Up	Apoptosis and oxidative stress	sirt1	[139]
miR-503	Rat	Up	Oxidative stress and apoptosis	nrf2	[140]
miR-326-3p	Mouse	Up	Metabolism and mitochondrial dysfunction	rictor	[141]
miR-30c	Mouse, rat	Down	Autophagy	beclin1	[142]
		Down	Hypertrophy and apoptosis	p53, p21	[143]
		Down	Hypertrophy	cdc42, pak1	[144]
		Down	Oxidative stress and apoptosis	pgc-1β	[145]
miR-133a	Mouse	Down	Fibrosis	erk1/2, smad-2	[146]
miR-200b	Mouse	Down	Cardiac fibrosis	p300	[147]
miR-222	Mouse	Down	Cardiac fibrosis	β-catenin	[148]
miR-551b-5p	Rat	Down	Autophagy	Protocadherin 17	[110]
miR-1	Rat	Down	Oxidative stress	Junctin	[149]
miR-9	/	Down	Pyroptosis and inflammation	elavl1 casp-1	[150]
miR-203	Mouse	Down	Oxidative stress, hypertrophy, fibrosis, and apoptosis	pik3ca	[151]
miR-21	Mouse	Down	Oxidative stress, hypertrophy, fibrosis, and apoptosis	Gelsolin, ppara, dusp8, spry1, ar	[152]
miR-22	Mouse	Down	Oxidative stress and apoptosis	sirt1	[153]
miR-373	Mouse	Down	Hypertrophy	mef2c	[154]
miR-15	Mouse	Down	Fibrosis	tgfbr1	[155]
miR141	Mouse	Down	Inflammation and fibrosis	nlrp3 and tgf-β1	[156]
miR146a	Mouse	Down	Inflammation and fibrosis	irak and traf6	[157]

MiRNAs regulate gene expression by binding to the 3’ UTR of different genes, implying that each may be involved in various pathologic processes of DCM. miRNA-30c expression levels were reduced in db/db mice, and its specific overexpression at the cardiac site ameliorated lipid accumulation, ROS generation, and apoptosis in cardiomyocytes. miRNA-30c can regulate myocardial metabolic disorder by binding to peroxisome proliferator-activated receptor-gamma coactivator-1 beta. In addition, miRNA-30c targets apoptosis-related genes, such as beclin1, p53, and p21, inhibiting diabetes-induced programmed cardiomyocyte death. The same downregulated miRNA133a in diabetic hearts is involved in cardiac remodeling. Combined with COL1A1, ERK1/2, and SMAD-2, miRNA133a suppresses collagen synthesis in the myocardial interstitium and cardiac fibrosis [90, 91]. Norepinephrine enhances the contractile capacity of the myocardium by activating beta receptors in cardiomyocytes. In DCM, β receptors are abnormally inactivated, and the contractile function of the heart is impaired. Nandi et al. [92] constructed miRNA-133a transgenic mice and unveiled that miRNA133a improved the contractile function of the diabetic heart by binding to the 3' UTR of tyrosine aminotransferase and promoted the synthesis of norepinephrine.

Several studies have confirmed the protective effects of miR-21 against cardiovascular diseases. miR-21 can improve fibrosis and apoptosis of cardiomyocytes; moreover, the hypoglycemic drug vildagliptin exerts hypoglycemic and cardioprotective effects through the miR-21/SPRY1/ERK/ mammalian target of rapamycin pathway [93]. The p38/ mitogen-activated protein kinase (MAPK) signaling pathway is significantly activated in diabetes and is involved in various pathological processes of DCM, such as oxidative stress, apoptosis, and ventricular remodeling [94]. HG-induced miR-21 overexpression activates the downstream p38/MAPK pathway and, thus, participates in ventricular remodeling in DCM [90].

Further, miR-320 is specifically expressed in the cardiomyocytes of DCM mice and can be detected in the plasma even before cardiac diastolic function is affected [95]. Knocking out miR-320 in DCM mice significantly improved glycolipid metabolism and cardiac function. This suggests that miR-320 is crucial in DCM and may be a potential target for its diagnosis and treatment. Unlike cytoplasmic miRNAs, nuclear miR-320 could bind to the promoter of the fatty acid receptor CD36 gene, leading to its expression.

## Potential clinical application of epigenetic regulators in DCM

### Epigenetic biomarkers

There are no obvious symptoms at the subclinical period of DCM, which makes detection and diagnosis more difficult. Serial studies have demonstrated epigenetic biomarkers play a vital impact on the early diagnosis and treatment of DCM over the last decade.

DNA methylation could be detected in blood and has been reported to be associated with the occurrence of cardiovascular diseases and diabetic complications. Hu et al. [96] identified that the hypomethylation of vascular endothelial growth factor (VEGFB), placental growth factor (PLGF), phospholipase C beta 1(PLCB1), and fatty acid transport protein 4 (FATP4) was associated with the incidence of diabetes with cardiovascular diseases, prompting that DNA methylation level might be a potential biomarker. Interestingly, in a new cross-sectional analysis, researchers found that increased DNA methylation age was related to cardiometabolic risk and worse cardiovascular prognosis, indicating the function of promising biomarkers [97]. In addition, Gadd et al. [98] utilized a machine learning strategy to construct a diabetes-associated epigenetic scores tool. The tool based on the genetic information carried by DNA methylation could depict methylation-proteomic features for diabetes prediction and risk stratification, including diabetic heart disease.

NcRNAs, especially miRNAs, have also emerged as potential lipid biopsy biomarkers in diabetic heart diseases owing to their availability and stability in biofluids. In a previous review by Jin, miRNAs targeting diabetes-associated cardiac fibrosis, which may act as potential biomarkers, had been summarized [99]. Here we mainly focused on the recently validated ncRNAs in human studies. Bielska A et al. indicated that five up-regulated miRNAs (miR-615-3p, miR-3147, miR-1224-5p, miR-5196-3p, and miR-6732-3p) in serum showed high diagnostic value (AUC > 0.8) for diabetic patients with ischemic heart disease [100]. Furthermore, in a 5-year prospective study, increasing cardiac hypertrophy of diabetic patients was paralleled by the up-regulation of miR122-5p, which was independent of glycemic control [101]. To further investigate the underlying mechanism, they constructed the diabetic mice model and found that miR122-5p was involved in diabetic cardiomyopathy by modulating extracellular matrix gene expression. These indicated the potential of miR-122 expression in evaluating the early stage of DCM, which was characteristic of subclinical diastolic dysfunction.

### Potential epigenetic therapies in DCM

In recent years, drug development based on several epigenetic regulatory molecules has improved the treatment of various diseases. Small-molecule inhibitors, such as azacitidine and decitabine, which target DNMTs and alleviate the inhibition of gene transcription due to methylation, have been applied in the treatment of myelodysplastic syndrome. Although drugs targeting enzymes involved in DCM remain unexplored, some recent discoveries may inspire research in this area. A real-world study showed that DNA methylation is associated with hypoglycemic drug response. Sonia et al. evaluated genome-wide DNA methylation in patients with type 2 diabetes mellitus and found that patients whose genomes showed greater methylation were more likely to tolerate metformin. They used combined weighted methylation risk scores based on 11 methylation sites to analyze the potential of DNA methylation to identify the risk of metformin tolerance, with the area (AUC) under the ROC more than 0.8 in different cohorts [102]. These results suggest that DNA methylation could serve as a predictive factor for medication evaluation. HDAC inhibitors have been developed in the clinical treatment of cancer. However, studies in the cardiovascular field remain in the preclinical stage. Travers et al. [31] found that HDAC inhibitors improved cardiac diastolic dysfunction. In their study, they constructed a diastolic insufficiency model via unilateral nephrectomy and injection of deoxycorticosterone acetate and found that the HDAC inhibitor ITF2357 could significantly inhibit cardiomyocyte fibrosis and ameliorate ventricular remodeling. Notably, diastolic dysfunction is a typical feature in the early stages of DCM. Therefore, conducting an in-depth study of HDAC inhibitors for the treatment of DCM holds great promise.

Studies on the application of BRD4 inhibitors in DCM treatment showed initial results. However, these studies were primarily conducted with animal models. In DCM mice, JQ1 significantly improved mitochondrial function, inhibited cardiomyocyte apoptosis and fibrosis, and improved diabetes-induced cardiac impairment [103]. Another BRD4 inhibitor, apaberon (APA), significantly ameliorated diabetic peripheral vascular damage. A recent large randomized double-blind clinical trial showed that the addition of APA to standard medical therapy did not significantly improve the incidence of major cardiovascular events in patients with acute coronary syndrome, type 2 diabetes, and low levels of high-density lipoprotein [104]. A subgroup analysis on the association between APA and type 2 diabetes remains lacking.

Given the prominent gene-silencing function of small RNAs in disease progression, RNA-based therapeutics have become a vital research direction for drug development. miR-10b-5p miRNA-targeted drugs act on pancreatic and fat cells to improve insulin resistance [105]. However, their safety, efficacy, and effects on DCM should be confirmed through further clinical studies.

### Epigenetic editing

The rapid advancement of epigenome editing technology from Zinc-finger, transcription activator-like effectors (TALEs) to the clustered regularly interspaced short palindromic repeats (CRISPR)-associated protein 9 (cas9) dCAS9 technique shows us a novel and breakthrough direction for the treatment of diseases [106, 107]. Though there was still no research focusing on DCM, the application of epigenetic editing in metabolic diseases did give us some inspiration. Ou et al. [108] investigated epigenome editing technology as a promising tool for inducing β cell proliferation previously. In their research, the methylation levels of the imprinting control region 2 (ICR2), which affected the expression of cell cycle inhibitor p57, were significantly reduced by TALE in pancreatic islets B cells. Recently, the dCAS9 system based on epigenetic regulatory factors precisely regulated disease-related genes while preserving the integrity of the genome. Matboli et al. [109] used CRISPR/CAS9 to knockout LncRNA-RP11-773H22.4 in peripheral blood mononuclear cells (PBMCs) of T2DM patients, and insulin resistance-related genes were altered significantly. Of note, the latest research published in *Nature* proposed a novel epigenome editing tool, EvoETR, with more powerful efficiency and specificity compared with CRISPR cas9 epi-silencing [60]. EvoETR-mediated PCSK9 inhibition in mice lasted for one year in mice, laying the foundation for effective in vivo therapeutics based on epigenetic editing.

Indeed, there are plenty of challenges to the application of epigenome editing in clinical practice, such as off-target and nonspecific effects. However, site-specific epigenetic modifications remain a very active area of translational research, warranting the need for more studies.

## Conclusions and future perspectives

DCM is a unique manifestation of systemic metabolic disorders caused by hyperglycemia or hyperlipidemia in the heart and is the most severe diabetic complication. In this review, we focused on the epigenetic regulation in DCM. First, we reviewed the basic epigenetic regulation patterns, including DNA methylation, histone modification, and ncRNAs. Then, we went to current investigations into the mechanisms of epigenetic regulation, which form a complex network that regulates gene expression at the transcriptional and post-transcriptional levels in DCM. Due to the feature of availability and stability in biofluids, a great number of epigenetic modifiers could serve as potential biomarkers for the early diagnosis and treatment of DCM. Although limited and remaining in the animal experimental stage, all available evidence about drugs targeting epigenetic regulators in DCM show that epigenetic modifiers hold great promise for the treatment of DCM.

It is noteworthy that issues and challenges exist in the mechanism investigation and clinical translation. Epigenetic modifications and ncRNAs play vital roles in metabolic memory. However, researches on the function of epigenetic modifications and ncRNAs underlying hyperglycemic memory are limited. Although current studies indicate strong therapeutic potential of epigenetic modifiers, few focus on patient data. In addition, in an era of high-throughput technology, it is likely to provide systematical insight and opportunities for effective therapy to combine multi-omics and single-cell sequencing techniques. Ongoing researches on the evolution and application of epigenetic editing therapy in DCM are also needed, which is expected to yield new insights into the pathogenesis and treatment of DCM.

## Data Availability

Not applicable.

## Abbreviations

α-MHC: α-myosin heavy chain
ADP: Adenosine diphosphate
AP: Activator protein
APA: Apaberon
BET: Bromodomain and extra-terminal domain
BRD4: Bromodomain protein 4
CRISPR: Clustered regularly interspaced short palindromic repeats
DCM: Diabetic cardiomyopathy
DNMT: DNA methyltransferase
ERK: Extracellular regulated protein kinases
EZH2: Enhancer of zeste homolog 2
FUS: Fused in sarcoma
GLUT: Facilitative glucose transporter
GPX1: Glutathione peroxidase 1
H3K27ac: Histone H3 lysine 27 acetylation
H3K27me3: Trimethylated histone H3 at lysine27
H3K9me3: Trimethylated histone H3 at lysine9
HATs: Histone acetyltransferases
HDACs: Histone deacetylase
HG: High glucose
HIF: Hypoxia-inducible factor
IL: Interleukin
JAK-STAT: Janus kinase-signal transducer and activator of transcription
lncRNA: Long non-coding RNA
MAPK: Mitogen-activated protein kinase
miRNA: MicroRNAs
ncRNA: Non-coding RNAs
NF-κB: Nuclear factor kappa-B
NLRP3: NOD-like receptor thermal protein domain-associated protein 3
PRC2: Polycomb repressive complex 2
ROS: Reactive oxygen species
SERCA2a: Sarcoplasmic/endoplasmic reticulum Ca^2+^ ATPase 2a
SIRT: Sirtuin
SOCS: Suppressor of cytokine signaling
STZ: Streptozotocin
TET: Ten-eleven translocation
TGF-β: Transforming growth factor beta
UTR: Untranslated region
